# A Single Oral Dose of Diclofenac Causes Transition of Experimental Subclinical Acute Kidney Injury to Chronic Kidney Disease

**DOI:** 10.3390/biomedicines10051198

**Published:** 2022-05-22

**Authors:** Johanna Störmer, Wilfried Gwinner, Katja Derlin, Stephan Immenschuh, Song Rong, Mi-Sun Jang, Nelli Shushakova, Hermann Haller, Faikah Gueler, Robert Greite

**Affiliations:** 1 Nephrology, Hannover Medical School, 30625 Hannover, Germany; johanna.m.stoermer@stud.mh-hannover.de (J.S.); gwinner.wilfried@mh-hannover.de (W.G.); rong.song@mh-hannover.de (S.R.); jang.mi-sun@mh-hannover.de (M.-S.J.); sushakova.nelli@mh-hannover.de (N.S.); haller.hermann@mh-hannover.de (H.H.); gueler.faikah@mh-hannover.de (F.G.); 2 Radiology, Hannover Medical School, 30625 Hannover, Germany; derlin.katja@mh-hannover.de; 3 Transfusion Medicine, Hannover Medical School, 30625 Hannover, Germany; immenschuh.stephan@mh-hannover.de; 4 Phenos GmbH, Feodor-Lynen-Str. 5, 30625 Hannover, Germany

**Keywords:** acute kidney injury, chronic kidney disease, ischemia–reperfusion injury, diclofenac nephrotoxicity

## Abstract

Nephrotoxic drugs can cause acute kidney injury (AKI) and analgesic nephropathy. Diclofenac is potentially nephrotoxic and frequently prescribed for pain control. In this study, we investigated the effects of single and repetitive oral doses of diclofenac in the setting of pre-existing subclinical AKI on the further course of AKI and on long-term renal consequences. Unilateral renal ischemia–reperfusion injury (IRI) for 15 min was performed in male CD1 mice to induce subclinical AKI. Immediately after surgery, single oral doses (100 mg or 200 mg) of diclofenac were administered. In a separate experimental series, repetitive treatment with 100 mg diclofenac over three days was performed after IRI and sham surgery. Renal morphology and pro-fibrotic markers were investigated 24 h and two weeks after the single dose and three days after the repetitive dose of diclofenac treatment using histology, immunofluorescence, and qPCR. Renal function was studied in a bilateral renal IRI model. A single oral dose of 200 mg, but not 100 mg, of diclofenac after IRI aggravated acute tubular injury after 24 h and caused interstitial fibrosis and tubular atrophy two weeks later. Repetitive treatment with 100 mg diclofenac over three days aggravated renal injury and caused upregulation of the pro-fibrotic marker fibronectin in the setting of subclinical AKI, but not in sham control kidneys. In conclusion, diclofenac aggravated renal injury in pre-existing subclinical AKI in a dose and time-dependent manner and already a single dose can cause progression to chronic kidney disease (CKD) in this model.

## 1. Introduction

Acute kidney injury (AKI) and chronic kidney disease (CKD) are closely interconnected [1,2]. On the one hand, AKI can progress to CKD [2]. On the other hand, chronic pre-existing renal damage, including not functionally relevant renal injury, is a risk factor for the development of AKI [2,3]. The aetiology of AKI is diverse [4,5,6,7,8] and a major cause of AKI is ischemia–reperfusion injury (IRI) which often occurs in post-operative settings [8].

Diclofenac, a non-steroidal anti-inflammatory drug (NSAID), is among the most commonly prescribed drugs and is available ‘over the counter’ in some countries [9,10]. It is typically used for relief of post-operative pain, trauma, or joint pain and acts through inhibition of cyclooxygenase (COX) 1 and 2, derivatives of the arachidonic acid cycle [11]. However, well-known side effects of diclofenac treatment are gastrointestinal bleeding and cardiovascular complications [12]. In addition, nephrotoxicity of diclofenac has been demonstrated experimentally in several ex vivo and in vivo models [13,14,15,16]. In humans, chronic diclofenac use was associated with increased AKI risk in the general and CKD populations [17,18]). Moreover, high dose NSAID use was associated with further renal function decline of up to 26% in CKD patients in a recent meta-analysis, although all NSAIDs were investigated as one group and the specific risk of diclofenac was not indicated [19]. Therefore, current studies link diclofenac to the development of AKI and to further renal function decline in CKD, but the role of diclofenac in AKI-to-CKD transition is not fully elucidated.

In this experimental study, we investigated the effects of single and repetitive oral doses of diclofenac on AKI and progression to CKD in the setting of pre-existing renal injury and hypothesized that a single oral dose of diclofenac is sufficient to cause AKI-to-CKD transition.

## 2. Materials and Methods

### 2.1. Animals

Male CD1 mice (8–10 weeks of age, body weight 30–35 g) purchased from Charles River (Sulzfeld, Germany) were used for the experiments. All mice had free access to drinking water and food. The day/night cycle was 14/10 h. All experiments were approved by the local animal protection committee of the Lower Saxony State department for animal welfare and food protection (14/1657 and 16/2295). Mice were monitored daily for their physical condition after surgery. Reasons for study termination were visible behavioral changes such as scrubby appearance, reduced motility, reduced food uptake, reduced activity, or body weight reduction of >20%. Unilateral IRI and sacrifice at day 1 was done in *n* = 5 mice each group. Bilateral renal IRI to study renal function was performed in *n*= 3 vehicle, *n =* 4 mice treated with 100 mg, and in *n =* 5 mice treated with 200 mg diclofenac. Unilateral IRI with long-term follow-up of two weeks was performed in *n =* 3 vehicle, in *n =* 5 mice treated with a single dose of 100 mg, and in *n =* 4 mice treated with 200 mg diclofenac. To study the effect of repetitive diclofenac treatment in the context of subclinical AKI *n =* 5 mice from each group were used.

### 2.2. Diclofenac Treatment

Diclofenac (Sigma-Aldrich, St. Louis, MO, USA) was dissolved in 15% polyethylenglycol 400 (PEG 400) (Sigma-Aldrich, St. Louis, MO, USA) and diluted in phosphate-buffered saline (PBS) (Th. Geyer, Renningen, Germany). Diclofenac was administered at a dose of 100 mg/kg or 200 mg/kg. The dose is referred to as 100 mg and 200 mg for convenience. The dose equivalents between humans and mice have to be calculated based on the body weight and the species-specific pharmacokinetic features. The pharmacokinetics in mice is 10 times higher than in humans [20,21]. This explains the apparent differences between the diclofenac doses commonly used in humans and the doses used in mice in this study. The diclofenac doses used here are in the range of those reported by other studies in mice [16,22].

Diclofenac treatment was performed via oral gavage immediately after the end of surgery (single dose) or on three consecutive days, starting immediately after surgery (repetitive treatment). Vehicle-treated mice received 15% PEG 400 diluted with PBS.

### 2.3. Ischemia Reperfusion Injury (IRI)

Isoflurane was administered for anesthesia (3% induction, 1–2% maintenance) and butorphanol (1 mg/kg) for analgesia. IRI was induced by unilateral or bilateral renal pedicle clamping with a microaneurysm clip (Aesculap, Germany) for 15 min. Reperfusion was controlled visually. Sham surgery was performed by opening of the abdominal cavity, but without manipulation of the renal vessels.

### 2.4. Determination of Blood Urea Nitrogen (BUN) Clearance

For determination of renal function by blood urea nitrogen (BUN), clearance bilateral renal IRI was performed. For all other experiments, unilateral renal IRI was conducted. One day after bilateral renal IRI, mice were placed into metabolic cages for a time period of 10 h and urine and blood were collected. BUN in urine and plasma was analyzed using the Olympus AU400 Chemistry Analyzer (Backman Coulter, CA, USA). BUN clearance was calculated using the following formula:

$$\mathrm{BUN}-\mathrm{clearance}\;\left[µL/\min\right]=1+\frac{\mathrm{urine}-\mathrm{BUN}\;\left[\frac{\mathrm{mmol}}{L}\right]\times \;\mathrm{urine}-\mathrm{volume}\;\left[µL\right]}{\mathrm{plasma}-\mathrm{BUN}\;\left[\frac{\mathrm{mmol}}{L}\right]\times \;\mathrm{time}\;\left[\min\right]}$$

### 2.5. Organ Preservation

Mice were sacrificed in deep general anesthesia (5% isoflurane) at 24 h, at three days and two weeks after IRI and organ retrieval was performed. After midline laparotomy, whole-body perfusion with ice-cold 0.9% PBS via the cannulated left ventricle resulted in a circulatory arrest. Organs were dissected and fixed in RNA later or 4% paraformaldehyde.

### 2.6. Renal Morphology and Immunofluorescence

After paraffin embedding, 2 μm sections were cut and stained with periodic acid Schiff (PAS) and Masson trichrome according to standard protocols. Immunofluorescence was performed with a fibronectin antibody (Abcam, Cambridge, UK) and a Gr-1 antibody for neutrophils (Ly-6G/Ly-6C+, Serotec, Oxford, UK). Morphological kidney damage was evaluated using an acute tubular injury (ATI) score assessing tubular dilatation, loss of brush border, necrosis, and vacuolization, and was expressed as a percentage of tubuli affected in the renal cortex and outer medulla. Interstitial fibrosis and tubular atrophy (IFTA) were scored semi-quantitatively and expressed as a percentage area of the total area. Renal fibronectin expression was determined and scored using the following semi-quantitative score: 0: <5% of area expressing fibronectin; 1: 6–25% of area expressing fibronectin; 2: 26–50% of area expressing fibronectin; 3: 51–75% of area expressing fibronectin; and 4: >75% of area expressing fibronectin. Renal neutrophil infiltration in the outer medulla (OM) of the kidney was analyzed semi-quantitatively using the following score: 0: <5 cells/view field (VF); 1: 5–10 cells/VF; 2: 11–20 cells/VF; 3: 21–50 cells/VF; and 4: >50 cells/VF. Analysis was conducted on a Leica imaging microscope at 200-fold magnification in 10 different view fields per sample. Investigators were blinded to the group assignment. Images were captured with the same magnification.

### 2.7. Cytokine Expression

Total mRNA was isolated from cross-sectioned kidney slices using RNeasy Mini Kit (Qiagen, Hilden, Germany) and cDNA was subsequently synthetized with Prime Script Reverse Transcriptase reagent (Takara, Kusatsu, Japan) from DNase-treated total RNA. A LightCycler 96 (Roche, Penzberg, Germany) was used to conduct qPCR. The following primers were used: connective tissue growth factor (CTGF) (Qiagen, #QT00096131), collagen 1 alpha 1 (col1a1) (Qiagen, #QT00162204), C-X-C motif chemokine 2 (CXCL2) (Qiagen, #QT00113253) and neutrophil gelatinase-associated lipocalin (NGAL) (Qiagen #QT00113407). Hypoxanthine phosphoribosyl transferase (HPRT) (Qiagen, #QT00166768) served as housekeeper for normalization.

### 2.8. Statistical Analysis

Statistical analysis was performed with GraphPad Prism (GraphPad Software, San Diego, CA USA, Version 5.0). Multiple comparisons were analyzed by one-way ANOVA and group means were compared using the Tukey’s post hoc test. Data is reported as mean value ± standard error of the mean (SEM). *p*-values < 0.05 were accepted as significant.

## 3. Results

### 3.1. Diclofenac Causes Aggravation of Renal Damage and Renal Function Impairment 24 h after IRI in a Dose-Dependent Manner 

#### 3.1.1. Renal Morphology 24 h after Unilateral IRI and a Single Oral Dose of Diclofenac

**Figure 1:**
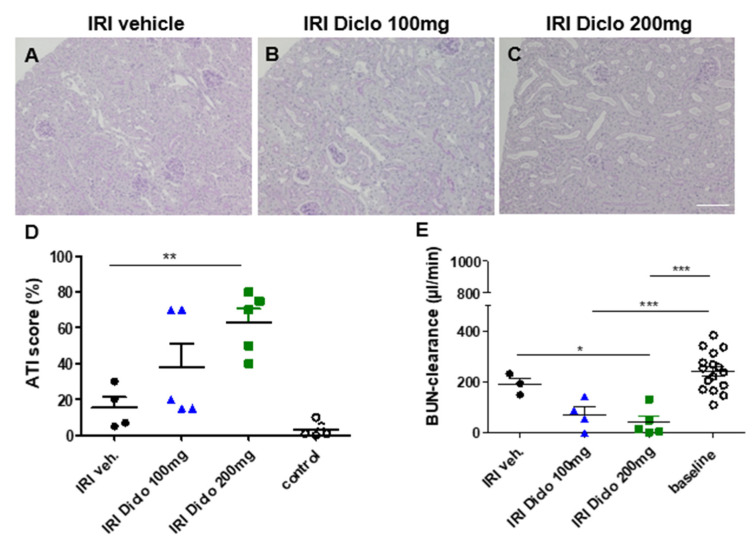
Renal morphology and function 24 h after renal ischemia reperfusion injury (IRI) and diclofenac treatment. PAS-stained kidney sections of IRI mice treated with vehicle (**A**), or a single oral dose of 100 mg diclofenac (**B**) or 200 mg diclofenac (**C**) are shown. Acute tubular injury (ATI) score is shown in (D). Diclofenac caused aggravation of renal damage in a dose-dependent manner. Renal morphology of contralateral control kidneys was comparable between groups and the untouched contralateral kidney of the IRI vehicle group is shown here as control. For evaluation of renal function, bilateral renal IRI was performed and urine of IRI mice treated with vehicle or a single dose of 100 mg or 200 mg diclofenac was collected in metabolic cages 24 h after IRI. Blood urea nitrogen (BUN) clearance was calculated and is shown in (E). A single oral dose of 200 mg diclofenac after IRI markedly worsened renal function. In the IRI groups, *n =* 3–5 mice are shown. Baseline BUN clearance was measured in *n =* 16 mice. * *p* < 0.05, ** *p* < 0.01, *** *p* < 0.001. Scale bar 100 µm.

We have previously shown that severity of AKI induced by IRI depends on the duration of the ischemia time [23]. To investigate whether a single oral dose of diclofenac already aggravates ischemic renal damage, a mouse model of mild transient renal ischemia of 15 min was used in this study. Acute tubular injury (ATI) was studied in the unilateral IRI model and the untouched contralateral kidney served as control. When diclofenac was administered in the setting of IRI, a dose-dependent aggravation of ATI was observed (Figure 1A–D). Treatment with vehicle or a single dose of 100 mg diclofenac after renal IRI only resulted in minor morphological kidney damage (Figure 1A,B,D). However, when a single dose of 200 mg diclofenac was administered after renal IRI, the ATI score as morphological correlate of AKI was markedly increased (Figure 1C,D). The contralateral control kidneys between groups revealed normal renal morphology, indicating that diclofenac only caused aggravation of renal damage in the setting of pre-existing injury in this model.

#### 3.1.2. Renal Function 24 h after Bilateral Renal IRI and a Single Oral Dose of Diclofenac

Evaluation of renal function is not feasible in the unilateral IRI model since the healthy contralateral kidney compensates for loss of renal function. Therefore, a bilateral renal IRI model with or without administration of a single diclofenac dose was applied and mice were placed into metabolic cages 24 h after IRI to collect urine and to calculate BUN clearance. In this subclinical model, bilateral IRI and vehicle treatment did not significantly reduce BUN clearance (Figure 1E). However, when diclofenac was administered in bilateral renal IRI, BUN clearance was significantly reduced 24 h after IRI compared with the baseline. A single oral dose of 200 mg diclofenac after renal IRI significantly reduced BUN clearance compared with the vehicle treatment.

### 3.2. A Single Oral Dose of Diclofenac Causes Chronic Renal Damage in the Setting of Subclinical AKI

#### 3.2.1. Interstitial Fibrosis and Tubular Atrophy (IFTA) Two Weeks after Unilateral IRI and a Single Dose of Diclofenac

**Figure 2:**
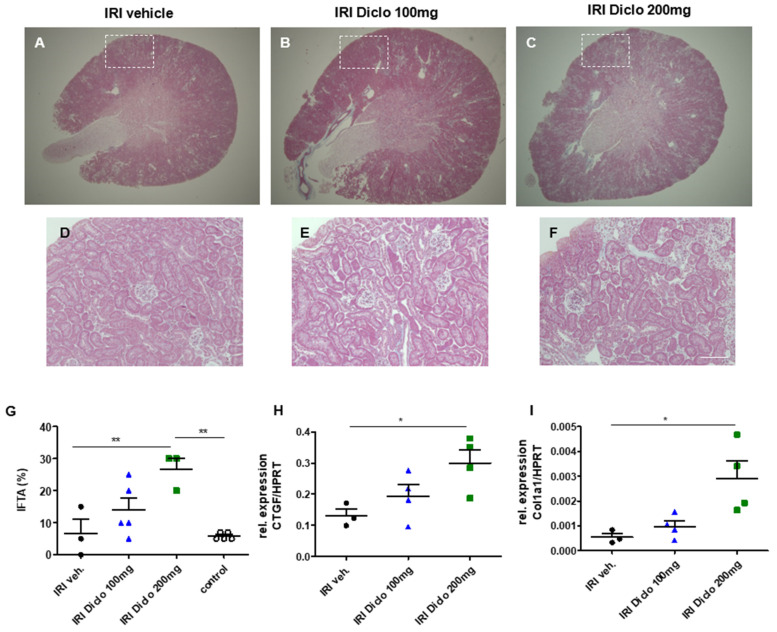
Chronic renal damage two weeks after renal ischemia–reperfusion injury (IRI) and diclofenac treatment. Masson trichrome-stained whole kidney sections of IRI mice treated with vehicle (**A**), or a single oral dose of 100 mg diclofenac (**B**) or 200 mg diclofenac (**C**) are shown. Interstitial fibrosis and tubular atrophy (IFTA) were scored in the renal cortex (white square, (**A**–**C**)). A single oral dose of 200 mg diclofenac in the setting of subclinical AKI caused enhanced IFTA after two weeks (**D**–**G**). Already 24 h after IRI, the pro-fibrotic markers, CTGF and collagen 1a1 (col1a1), were significantly elevated in the renal tissue in mice treated with a single dose of 200 mg diclofenac (**H**,**I**). In the IRI groups, *n* = 3–5 mice are shown. * *p* < 0.05, ** *p* < 0.01, Scale bar 100 µm.

To investigate whether a single oral dose of diclofenac administered in the setting of subclinical AKI already causes progression to chronic renal damage, unilateral IRI was performed and IFTA was determined two weeks after renal IRI and vehicle treatment or single-dose diclofenac treatment, respectively (Figure 2A–G). Vehicle treatment after IRI did not result in significant IFTA after two weeks (Figure 2A,D,G). However, oral administration of diclofenac in the setting of subclinical AKI caused increased IFTA in a dose-dependent manner. There was a trend toward increased IFTA two weeks after IRI and a single dose of 100 mg diclofenac which did not reach statistical significance (Figure 2B,E,G). However, 200 mg diclofenac after IRI significantly increased IFTA as a hallmark of chronic renal damage after renal IRI (Figure 2C,F,G). The untouched contralateral control kidneys did not exhibit significant IFTA in all groups.

#### 3.2.2. Pro-Fibrotic Cytokines in the Renal Tissue 24 h after IRI

To investigate whether there are early indicators for the development of chronic renal damage, the pro-fibrotic markers CTGF and collagen 1 alpha 1 (coll1a1) were measured in the renal tissue 24 h after renal IRI and vehicle or a single dose of diclofenac treatment, respectively. A single dose of 200 mg, but not 100 mg, of diclofenac caused increased renal tissue expression of the pro-fibrotic markers CTGF (Figure 2H) and col1a1 (Figure 2I) 24 h after renal IRI.

### 3.3. Acute and Chronic Renal Effects of Repetitive Diclofenac Treatment in Subclinical AKI

#### 3.3.1. Renal Morphology and Tubular Injury Three Days after Renal IRI and Repetitive Diclofenac Treatment 

So far in this study, a single oral dose of 200 mg, but not 100 mg, of diclofenac caused chronic renal damage in the setting of pre-existing subclinical AKI. Next, renal effects of repetitive administration of 100 mg diclofenac over three days subsequent to renal IRI are investigated.

**Figure 3:**
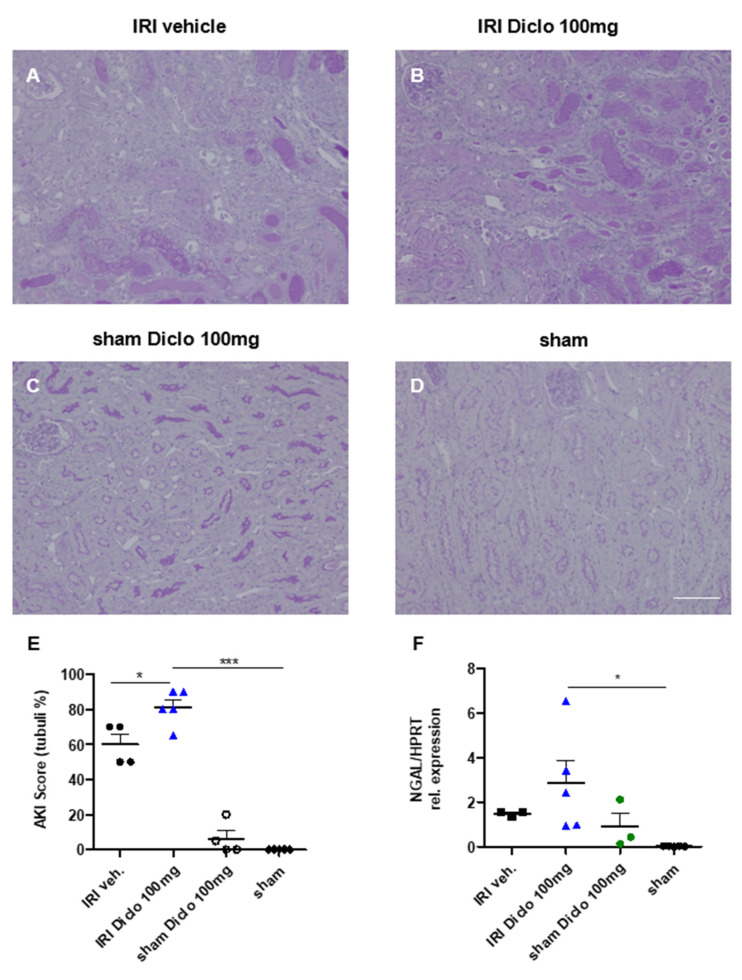
Renal morphology three days after renal ischemia–reperfusion injury (IRI) and repetitive diclofenac treatment. PAS-stained kidney sections of IRI mice treated with vehicle (**A**), or repetitive doses of 100 mg diclofenac over three days (**B**) are shown and acute tubular injury (ATI) as a morphological sign of AKI was assessed in the outer medulla. Sham-operated mice treated with 100 mg of diclofenac over three days (**C**) and vehicle-treated sham mice (**D**) served as control. Quantification of ATI is shown in E. Repetitive doses of diclofenac significantly aggravated morphological renal damage compared with vehicle treatment after IRI. Diclofenac treatment in the setting of sham surgery did not cause significant renal damage (**E**). Renal tissue NGAL expression increased after IRI and repetitive diclofenac doses indicating tubular injury (**F**). *n =* 3–5 mice in each group are shown. * *p* < 0.05, *** *p* < 0.001. Scale bar 100 µm.

Repetitive diclofenac treatment after mild renal IRI significantly aggravated renal injury (Figure 3A,B,E). To investigate whether a cumulative toxic effect of diclofenac also affects healthy kidneys, sham-operated mice treated with 100 mg diclofenac over three days served as controls in these experiments. Notably, repetitive diclofenac treatment did not cause renal injury in sham controls (Figure 3C,D,E).

Renal tissue levels of the injury marker NGAL were determined by qPCR 72 h after renal IRI and repetitive vehicle or diclofenac treatment, respectively. Subclinical AKI and vehicle treatment did not cause major NGAL upregulation, whereas repetitive diclofenac treatment after renal IRI resulted in marked renal NGAL upregulation as a tubular injury marker (Figure 3F).

#### 3.3.2. Neutrophil Infiltration in Diclofenac Treatment after Renal IRI

**Figure 4:**
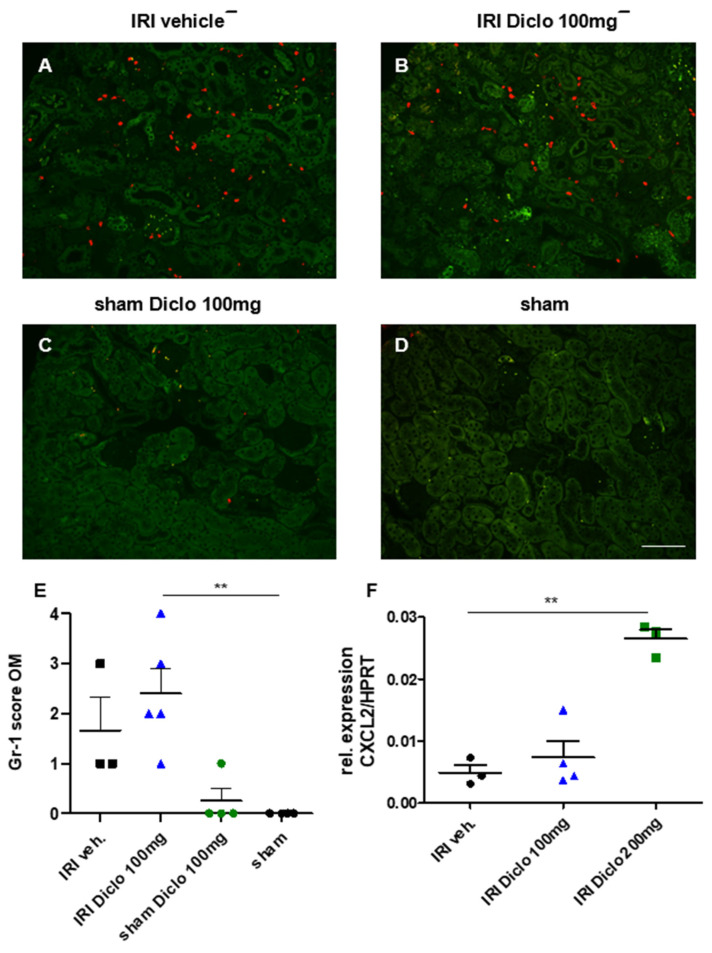
Renal neutrophil infiltration three days after renal ischemia–reperfusion injury (IRI) and repetitive diclofenac treatment and CXCL2 expression after a single oral dose of diclofenac. Gr-1 stain for neutrophils was performed on kidney sections of IRI mice treated with vehicle (**A**), or repetitive doses of 100 mg diclofenac (**B**) over three days are shown. Sham-operated mice treated with 100 mg diclofenac over three days (**C**) and vehicle-treated sham mice (**D**) served as control. Semi-quantitative scoring of renal neutrophil infiltration is shown in E. Repetitive doses of diclofenac after IRI significantly increased renal neutrophil infiltration. Diclofenac treatment in the setting of sham surgery did not cause significant neutrophil infiltration compared with sham surgery with vehicle treatment (**E**). In line, CXCL2, the chemoattractant for neutrophils, is upregulated in the kidney tissue in subclinical AKI and single-dose diclofenac treatment in a dose-dependent manner (F). *n =* 4–5 mice in each group are shown. ** *p* < 0.01. Scale bar 100 µm.

Gr-1 was stained to identify infiltrating neutrophils in the renal outer medulla three days after renal IRI and repetitive diclofenac treatment. Repetitive doses of 100 mg diclofenac over three days following renal IRI significantly enhanced renal neutrophil infiltration, whereas diclofenac treatment in the setting of sham surgery did not cause major neutrophil infiltration (Figure 4A–E). In line with this finding, CXCL2, the chemoattractant for neutrophils was already upregulated after a single dose of diclofenac in a dose-dependent manner (Figure 4F).

#### 3.3.3. Early Upregulation of Fibronectin in Repetitive Diclofenac Treatment after Renal IRI

**Figure 5:**
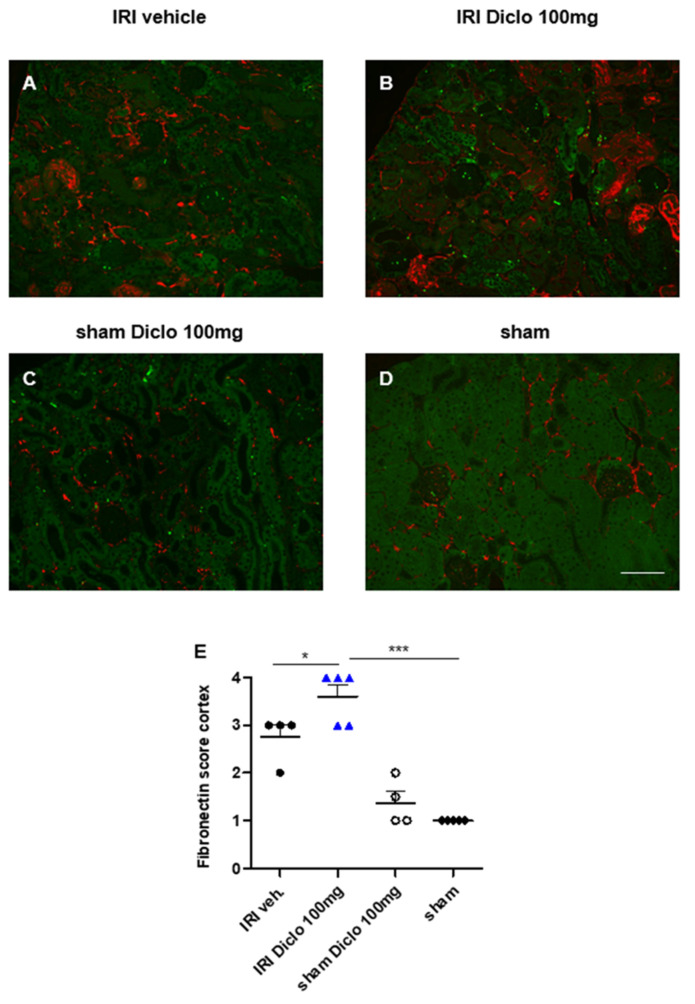
Renal fibronectin expression three days after renal ischemia–reperfusion injury (IRI) and repetitive diclofenac treatment. Fibronectin-stained kidney sections of IRI mice treated with vehicle (**A**), or repetitive doses of 100 mg diclofenac (**B**) over three days are shown. Sham-operated mice treated with 100 mg diclofenac over three days (**C**) and vehicle-treated sham mice (**D**) served as control. Semi-quantitative scoring of renal fibronectin expression is shown in (**E**). Repetitive doses of diclofenac after IRI significantly increased renal fibronectin expression as an early pro-fibrotic indicator after three days. Diclofenac treatment in the setting of sham surgery did not cause significant renal fibronectin expression compared with sham surgery with vehicle treatment (E). *n =* 4–5 mice each group are shown. * *p* < 0.05, *** *p* < 0.001. Scale bar 100 µm.

Expression of fibronectin in the renal cortex was determined three days after renal IRI and vehicle or repetitive diclofenac treatment. Repetitive doses of 100 mg diclofenac over three days following renal IRI significantly enhanced renal fibronectin expression compared with vehicle treatment (Figure 5A,B,E). Sham mice with or without repetitive diclofenac treatment over three days did not exhibit major renal fibronectin expression (Figure 5C,D,E).

Taken together, the findings indicate that diclofenac aggravated renal injury in the setting of pre-existing subclinical AKI in a dose- and treatment-duration-dependent manner. A single oral dose of 200 mg, but not 100 mg, of diclofenac administered after mild renal IRI aggravated AKI and caused progression to chronic renal damage. Repetitive diclofenac doses of 100 mg enhanced early renal injury and fibrosis in the setting of pre-existing subclinical AKI, whereas no effect was observed on healthy kidneys.

## 4. Discussion

Diclofenac treatment has been linked to increased AKI risk in the general population [17] and in CKD patients [18]. However, the mechanistic role of diclofenac in the vulnerable phase of AKI-to-CKD transition has not been studied.

In this study, we investigated the effect of diclofenac treatment (single or repetitive) on the progression of AKI-to-CKD in a mouse model of pre-existing subclinical AKI.

Previous studies have shown that the duration of ischemia time in murine renal IRI correlates with severity of subsequent AKI development and even determines whether AKI recovers or progresses to CKD [23,24]. Renal IRI for 35 min in CD1 mice induced overt AKI and progressive renal fibrosis after two weeks [23]. In this study, a 15 min renal IRI model was used to induce subclinical AKI characterized by minor morphological kidney damage without functional relevance and without progression to CKD.

However, additional diclofenac treatment aggravated early renal injury in a dose- and time-dependent manner. A single oral dose of 200 mg, but not 100 mg, of diclofenac reduced renal function and aggravated early morphological kidney damage in the setting of pre-existing subclinical AKI. In a previous study, a single oral dose of diclofenac caused marked nephrotoxicity involving oxidative stress and apoptosis [16]. An oxidative stress response has also been detected following repetitive doses of 100 mg diclofenac over three days [22]. Mechanistically, diclofenac inhibits prostaglandin synthesis via inhibition of cyclooxygenase 1 and 2, ultimately leading to renal vasoconstriction [11,25,26]. This vasoconstriction can facilitate renal ischemia and could be a possible explanation for aggravated tubular injury after diclofenac treatment in subclinical AKI. Consistently, diclofenac reduced renal perfusion measured by functional MRI [27] and contrast-enhanced ultrasonography (CEUS) in healthy individuals [28]. In line with our findings, diclofenac treatment at a dose of 200 mg induced AKI in a previous experimental study, even though this was in the absence of pre-existing renal damage [15]. In our study, the contralateral control kidney in the unilateral IRI model was also exposed to the single dose of diclofenac and revealed normal morphology indicating that the nephrotoxic effect of diclofenac administration only affected the pre-injured ischemic kidney. A possible explanation for these differences between the study by Bao and colleagues and our study could be strain-specific differences in the vulnerability to renal injury which have been described previously [23,29,30]. The increased susceptibility of pre-damaged kidneys to AKI is well recognized clinically and experimentally [31]. Diabetic or hypertensive patients with pre-existing CKD have an increased risk for hospitalization with AKI of diverse aetiology [32]. Similarly, impaired baseline renal function is a relevant risk factor for the development of AKI in patients undergoing major surgery [33]. In experimental models of CKD, such as renal mass reduction or nephrectomy, recovery from ischemic AKI is impaired and renal fibrosis development enhanced [31,34]. However, these findings are not fully transferable to our study since we did not use a CKD-mouse model, but our model started with normal, healthy kidneys and combined two injury factors, which were both unable to cause relevant renal injury on their own.

Maladaptive repair after AKI with development of tubular atrophy and progressive renal fibrosis is a hallmark of AKI-to-CKD transition [35]. Renal IRI can cause cell cycle arrest in tubular cells which in turn produce pro-fibrotic cytokines such as TGF-ß and the downstream target CTGF [36], ultimately leading to development of post-ischemic kidney fibrosis [8,37]. Activation of TGF-β signaling may prevent tubular cells from differentiation and regeneration after AKI [31,38]. Indeed, CTGF was upregulated already 24 h after a single dose of 200 mg diclofenac in IRI mice and marked interstitial fibrosis and tubular atrophy developed two weeks later in diclofenac, but not vehicle-treated IRI mice. In line with these findings, renal upregulation of CTGF 24 h after injury has been described as an early indicator for subsequent kidney fibrosis development in models of unilateral ureteral obstruction [39] and severe IRI [23]. Therefore, our results indicate that a single oral dose of 200 mg diclofenac shifted the continuum between AKI and CKD towards maladaptive repair with activation of TGF-β signaling which is well known to promote fibrosis in CKD [31,40].

In contrast to administration of 200 mg diclofenac, a single oral dose of 100 mg diclofenac did not aggravate AKI and did not cause AKI-to-CKD transition. To investigate whether this is a matter of treatment duration, sham-operated and IRI mice were subsequently treated with 100 mg diclofenac over three days. Repetitive doses of 100 mg diclofenac had no effect on renal morphology in sham-operated animals. Overt aggravation of tubular injury along with severe upregulation of the pro-fibrotic marker fibronectin in the kidney tissue was observed in the setting of pre-existing subclinical ischemia-induced AKI. Inhibition of fibronectin polymerization after experimental ischemic AKI has been shown to attenuate progressive renal fibrosis development in recent work [41], indicating that fibronectin is a potential therapeutic target in the prevention of diclofenac-associated transition of AKI to CKD.

A limitation of our study is the small sample size and that we did not directly measure plasma diclofenac levels or renal prostaglandin levels to determine target engagement in the renal IRI model. However, a previous study in mice found that plasma diclofenac levels markedly increase 30 min after administration of a single 200 mg dose even though effects of this single diclofenac dose on the kidney have not been investigated [42]. Future studies monitoring diclofenac plasma levels and measuring renal prostaglandin levels in larger cohorts with different diclofenac doses should determine renal target engagement in pre-injured kidneys.

The novel finding of this study is that a single oral dose of diclofenac is sufficient to cause AKI-to-CKD transition via early upregulation of pro-fibrotic factors in pre-injured, but not healthy kidneys. In conclusion, even a single oral dose of diclofenac should be avoided in the setting of pre-existing renal damage. 

## Data Availability

The data presented in this study are available on request from the corresponding author.
